# Yields of Photo-Proton Reactions on Nuclei of Nickel and Separation of Cobalt Isotopes from Irradiated Targets

**DOI:** 10.3390/molecules27051524

**Published:** 2022-02-24

**Authors:** Andrey G. Kazakov, Julia S. Babenya, Taisya Y. Ekatova, Sergey S. Belyshev, Vadim V. Khankin, Alexander A. Kuznetsov, Sergey E. Vinokurov, Boris F. Myasoedov

**Affiliations:** 1 Radiochemistry Laboratory, Vernadsky Institute of Geochemistry and Analytical Chemistry, The Russian Academy of Sciences, Kosygin St., 19, 119991 Moscow, Russia; fanta2000-10@mail.ru (J.S.B.); ekatova.t@gmail.com (T.Y.E.); vinokurov.geokhi@gmail.com (S.E.V.); bfmyas@mail.ru (B.F.M.); 2 Department of Physics, Lomonosov Moscow State University, Leninskie Gory, 1, Bld. 2, 119991 Moscow, Russia; belyshev@depni.sinp.msu.ru (S.S.B.); kuznets@depni.sinp.msu.ru (A.A.K.); 3 Skobeltsyn Institute of Nuclear Physics, Lomonosov Moscow State University, Leninskie Gory, 1, Bld. 2, 119991 Moscow, Russia; v-k32@yandex.ru

**Keywords:** photonuclear method, cobalt isotopes, cobalt-55, extraction chromatography, nuclear medicine

## Abstract

Nowadays, cobalt isotopes ^55^Co, ^57^Co, and ^58m^Co are considered to be promising radionuclides in nuclear medicine, with ^55^Co receiving the most attention as an isotope for diagnostics by positron emission tomography. One of the current research directions is dedicated to its production using electron accelerators (via photonuclear method). In our work, the yields of nuclear reactions occurring during the irradiation of ^nat^Ni and ^60^Ni by bremsstrahlung photons with energy up to 55 MeV were determined. A method of fast and simple cobalt isotopes separation from irradiated targets using extraction chromatography was developed.

## 1. Introduction

Two main methods for producing radioisotopes or their generators for nuclear medicine are widely used today: in nuclear reactors and cyclotrons. Another possible way of their production is photonuclear method. Production of radioactive isotopes for different purposes by this method was widely investigated in the 1970–1980s, and today the growing number of studies on medical isotopes production by photonuclear method can be observed. Due to the development of this method, nowadays ^47^Sc, ^67^Cu, and ^99^Mo/^99m^Tc generator as well as light isotopes ^11^C, ^13^N, ^15^O, ^18^F for positron emission tomography (PET) are already obtained in electron accelerators on a regular basis, and the production of ^225^Ac, ^177^Lu, ^111^In, ^105^Rh, and ^44^Ti/^44^Sc generator is currently being investigated [1].

Radioactive isotopes ^55^Co, ^57^Co, and ^58m^Co are considered to be used in nuclear medicine, as they are not too well known and well-studied but are promising. The most attention is paid to ^55^Co (T_1/2_ = 17.5 h, 77% β^+^, E_maxβ_^+^ = 1498 keV), which is auspicious for studying slow processes in an organism by PET. At the dawn of nuclear medicine, it was reported that ^55^Co complexes could be used for the diagnostics of lung cancer and for the visualization of tumors [2,3,4]. It was shown in contemporary studies that ^55^Co-EDTA is suitable for use in nephrological research [5] and for the visualization of prostate cancer [6] as well as for the detection of distant metastasis in close vicinity of the bladder and kidneys [7]. It is important to mention that the chemical properties of ^55^Co(II) and its behavior in an organism are similar to that of PET-isotope ^64^Cu(II) (T_1/2_ = 12.7 h, 17.4% β^+^, E_maxβ_^+^ = 653 keV) and also of Ca(II), the latter being present in body but lacking suitable radioactive isotopes for its visualization [8,9,10]. In comparison to ^64^Cu(II), ^55^Co has the following advantages: first, compounds labeled with ^55^Co tend to be absorbed less by the liver than compounds labeled with ^64^Cu. Second, the higher yield of positrons produced by ^55^Co results in less activity of the drug and/or less time required for PET diagnostics. Using ^55^Co in PET as an indicator of calcium allows one to visualize the affected tissue in patients with traumatic brain injury and to estimate neuronal damage with strokes and brain tumors [11,12,13]. As for other medical isotopes of Co, ^58m^Co (T_1/2_ = 9.04 h) is Auger emitter and thus is suitable for Auger therapy, and ^57^Co (T_1/2_ = 271.8 d, E_γ_ = 122 keV) is suitable for preclinical research and the study of pharmacokinetics of drugs based on cobalt due to the long half-life of this isotope and the high yield of produced gamma-quanta [14].

**Figure 1:**
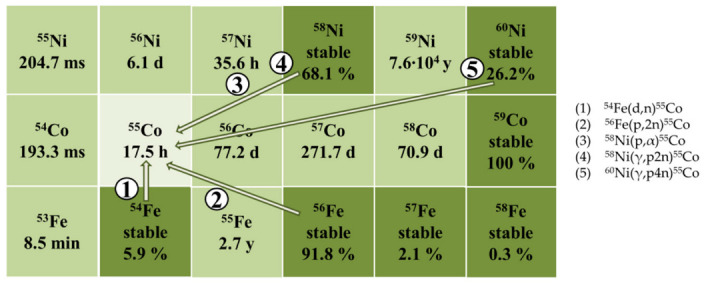
Studied methods of ^55^Co production.

In spite of the advantages listed above, the use of cobalt isotopes in medicine is limited by the difficulties of their production (Figure 1). ^55^Co is mainly produced in cyclotrons by nuclear reactions ^54^Fe(d,n)^55^Co, ^56^Fe(p,2n)^55^Co, and ^58^Ni(p,α)^55^Co [15]. However, all listed ways of production require enriched targets, which would also prevent long-lived radioactive impurities ^56^Co (T_1/2_ = 77.27 d) and ^57^Co from forming [16]. According to calculations, when a target made of 100% ^54^Fe is irradiated with deuterons, a yield of up to 30 MBq/μA·h can be achieved, while the content of long-lived cobalt isotopes is minimal [15]. In the case of irradiation of 100% ^56^Fe with protons, a significantly higher yield can be achieved up to 180 MBq/μA·h; however, the content of ^56^Co will also be higher. Finally, for the ^58^Ni(p,α)^55^Co reaction using an enriched target, the maximum yield is 13 MBq/μA·h, and the impurity content is minimal. Thus, ^54^Fe(d,n)^55^Co is the most promising reaction for use in nuclear medicine among cyclotron ones.

Cobalt isotopes (including ^55^Co) can also be obtained using an electron accelerator—by irradiation of nickel. Currently, data on yields of photo-proton reactions on nickel nuclei, leading to the formation of medical isotopes of cobalt, is limited. In works [17,18], flux-weighted average cross-sections of reactions ^nat^Ni(γ,pxn) in energy range of 55 to 75 MeV were determined. It was established that cross-sections of ^nat^Ni(γ,pxn)^55^Co in this range varied insignificantly. However, there are no data on the yields of nuclear reactions in these works, which makes it impossible to evaluate the possibility of obtaining cobalt isotopes in sufficient quantities for nuclear medicine using electron accelerators.

No methods of separation of cobalt isotopes produced in an electron accelerator can be found in the literature. At the same time, there are works dedicated to the separation of cobalt isotopes from cyclotron-irradiated nickel targets. In these works, Ni(II) and Co(II) were separated using anion exchange resin Dowex AG-1X8 [11,14,19,20,21,22,23,24,25,26,27] and using extraction chromatography sorbent based on diglycolamide, where Co(II) was obtained in 3 M HCl [28]. The best results were achieved in the last case, the yield of cobalt was 92%, separation factors of cobalt from different impurities varied from 8∙10^2^ to 2∙10^4^, and the process lasted for 2 h. It is worth mentioning that the main task of these works was more difficult than just separation of cobalt and nickel: first, irradiation of nickel also results in the formation of copper isotopes; second, nickel was usually applied via electrodeposition as a coating to a metal plate, irradiation of which also led to impurities. To produce cobalt isotopes for nuclear medicine purposes, it is necessary to develop a technique with higher yield, higher Ni/Co separation factors, and less time of separation, allowing one to obtain cobalt in diluted HCl medium.

Thus, the purpose of this work was to determine yields of photonuclear reactions on nickel nuclei and also to develop a fast, simple, and effective method of carrier-free cobalt isotopes separation from nickel targets.

## 2. Results and Discussion

### 2.1. Radionuclide Composition of Irradiated Targets and Yields of Nuclear Reactions

**Figure 2:**
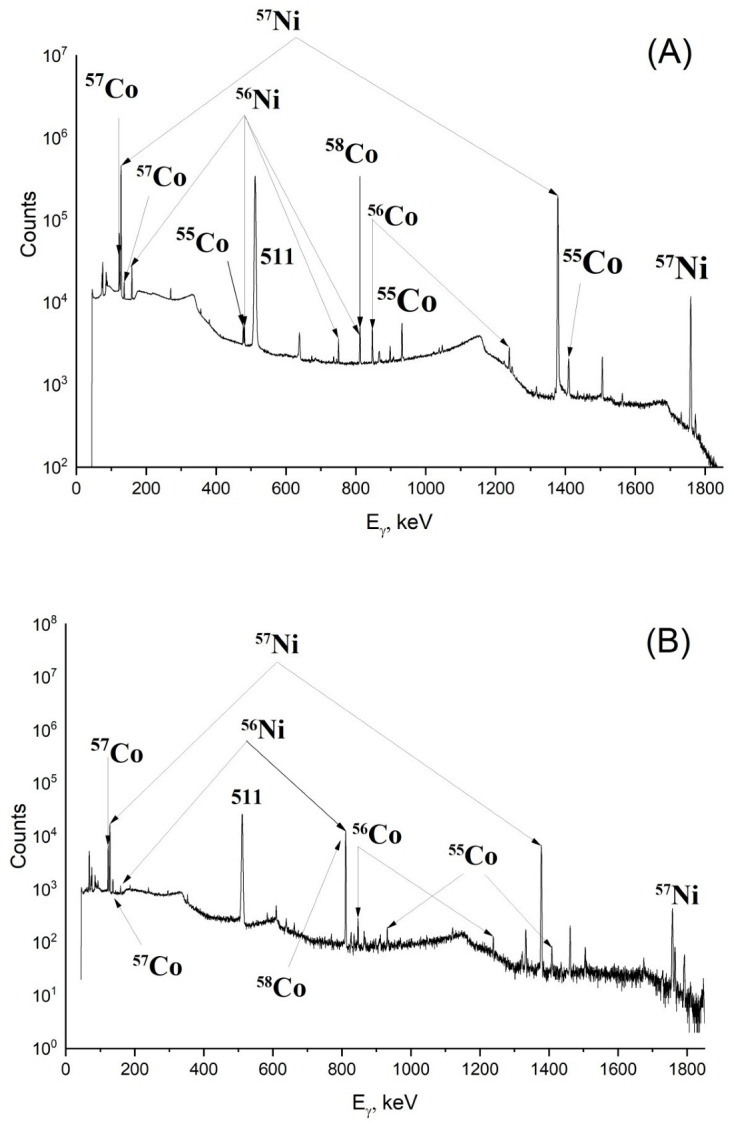
Gamma-spectra of ^nat^Ni irradiated by bremsstrahlung photons with energy up to 55 MeV during 1 h after EOB (**A**), and of irradiated ^60^Ni for 19 h after EOB (**B**). The most intense peaks of each isotope are labeled in the figures.

Gamma-spectra of irradiated targets made of ^nat^Ni and ^60^Ni are presented in Figure 2A,B; yields of photonuclear reactions leading to the formation of nickel and cobalt isotopes during the irradiation of ^nat^Ni, ^60^Ni, and ^58^Ni are presented in Table 1. It was established that in each case, ^55^Co was produced along with significant quantities of long-lived impurities ^56,57,58^Co. Irradiation of ^nat^Ni and ^58^Ni resulted in the yield of ^56,57,58^Co being 1.2–1.5 times higher than the yield of ^55^Co, and the irradiation of ^60^Ni led to the yield of ^55^Co being no more than 3% of the yield of all cobalt isotopes. Obviously, ^55^Co with such low radionuclide purity is not suitable for PET. On the other hand, isotopes ^56,57,58^Co are gamma-emitters and can be used in preclinical research of radiopharmaceuticals based on cobalt, including in vivo experiments. For these purposes, we recommend irradiating ^nat^Ni; in this case, the yield of ^57^Co is 66 kBq/(µA·h·g/cm^2^) on a thin plate, and this value can be increased by using massive target. ^58^Ni can also be used as a target material increasing the yield of ^57^Co up to 82 kBq/(µA·h·g/cm^2^) on thin foil; however, targets made of ^58^Ni are more expensive than ones made of ^nat^Ni. Therefore, photonuclear method allows one to produce cobalt isotopes with sufficient activity for preclinical research.

Table 1 also compares the experimentally measured yields with theoretical calculations using the TALYS program, taking into account the bremsstrahlung spectrum. On the whole, we can see a satisfactory agreement between the experimental yields and the theoretical calculations. The difference in values can be due to two main factors: TALYS uses default photoabsorption cross-sections, and also does not take into account the isospin splitting of the giant dipole resonance, which has a significant effect on the yields of photo-proton reactions.

### 2.2. Separation of Co(II) Isotopes without a Carrier Using Extraction Chromatography

**Figure 3:**
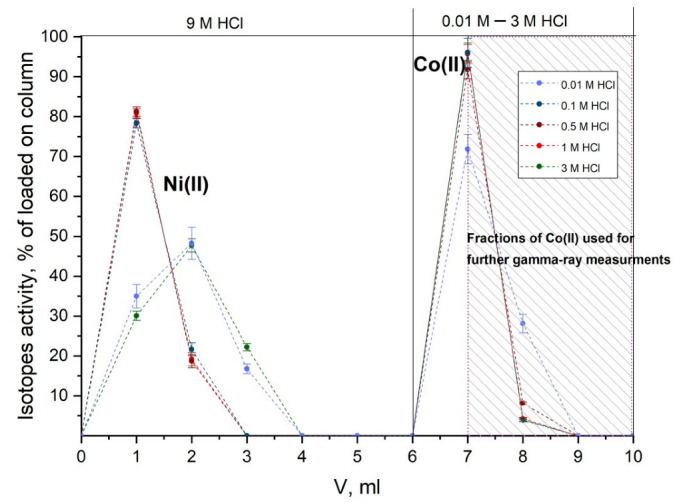
Elution curves of Ni(II) and Co(II) during separation on DGA resin column in HCl solutions.

Obtained chromatograms of Ni(II) and Co(II) separation using DGA resin are presented in Figure 3. It was established that elution of Co(II) by HCl solutions with concentration varying from 0.01 to 3 M resulted in similar elution profiles; yield of Co(II) was close to quantitative in each case, and the process lasted for no longer than 0.5 h. To determine the separation factor of Ni(II) and Co(II), fractions marked in Figure 3 were selected for gamma-spectra registration for 24 h. Peaks of isotopes ^56,57^Ni were absent in registered gamma-spectra, and the separation factor of Ni/Co was calculated using detection limit of these radionuclides and was 2.8·10^5^, which is one order of magnitude higher than the factor during the separation using sorbent with similar composition in work [28]. Thus, the method of carrier-free Co(II) separation was developed; it allows one to obtain necessary isotopes in different solutions of HCl, including the solutions with low concentration, which is preferable for nuclear medicine.

As stated above in Section 2.1, the highest yields of cobalt isotopes are achieved during the irradiation of enriched ^58^Ni targets. Obviously, the expensive target material should be regenerated after separation. For this purpose, it is possible to evaporate eluate-containing Ni(II) to dryness to irradiate NiCl_2_ to produce cobalt isotopes one more time. In this case, there will be no need to dissolve the target during prolonged heating, since NiCl_2_ is water-soluble. Another possible way is the irradiation of Ni(II) solution separated from the column, which can be eluted through the column again without any preparative treatment. In any case, the regeneration of enriched nickel is not complicated. It is also worth noting that formed isotopes of cobalt have T_1/2_ no longer than 272 d, allowing one to utilize samples with general waste after prolonged storage, i.e., no radioactive waste requiring special treatment and disposal is produced after the irradiation. Thus, this studied method of production and separation of cobalt isotopes is environmentally friendly due to the regeneration of the target and the absence of radioactive waste.

## 3. Materials and Methods

### 3.1. Theoretical Calculations of Cross-Sections and Yields of Photonuclear Reactions

**Figure 4:**
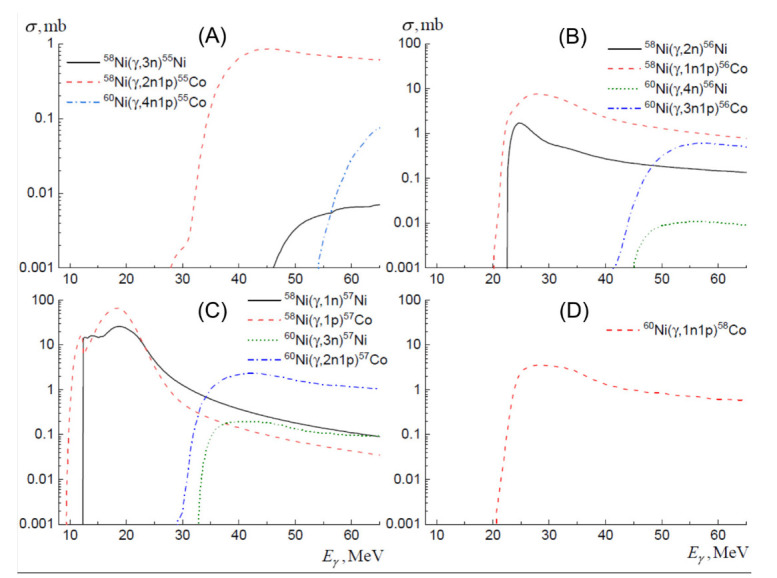
Calculated cross-sections of the main photonuclear reactions leading to the formation of ^55^Co and ^55^Ni (**A**), ^56^Co and ^56^Ni (**B**), ^57^Co and ^57^Ni (**C**), and ^58^Co (**D**).

The cross-sections were calculated using the TALYS program, while the total photoabsorption cross-section was calculated based on the parameters from the RIPL-2 experimental database [30]. To calculate the cross-sections for photonuclear reactions, TALYS uses a combination of the evaporative and exciton preequilibrium decay mechanism of a compound nucleus with the emission of nucleons and gamma-quanta. The obtained cross-sections of the main photonuclear reactions leading to the formation of ^55,56,57,58^Co and ^55,56,57^Ni isotopes are shown in Figure 4.

The theoretical yield of isotope formation, taking into account all possible reactions leading to the formation of the selected isotope, was calculated by Equation (1):

(1) $$Y=\frac{\lambda \alpha }{\rho q_{e}}\sum_{i}\eta_{i}\;\int_{E_{i}}^{E_{m}}\phi (E_{\gamma },E_{m})\sigma_{i}\left(E_{\gamma }\right)dE_{\gamma }$$

where *λ* is the decay constant, *α* is the number of studied nuclei per 1 cm^2^ of target, *ρ* is the surface density of the target, *q_e_* is the electron charge in µA·h, index *i* corresponds to the number of the reaction contributing to the formation of studied isotope, *η_i_* is the percentage of the nickel isotope on which the reaction occurs in a natural mixture of isotopes, *E_i_* is the threshold of the corresponding reaction, *E_m_* is the maximum energy of the bremsstrahlung spectrum, *σ_i_*(*E_γ_*) is the cross-section of the corresponding photonuclear reaction, and *ϕ*(*E_γ_*,*E_m_*) is the bremsstrahlung spectrum on the target.

**Figure 5:**
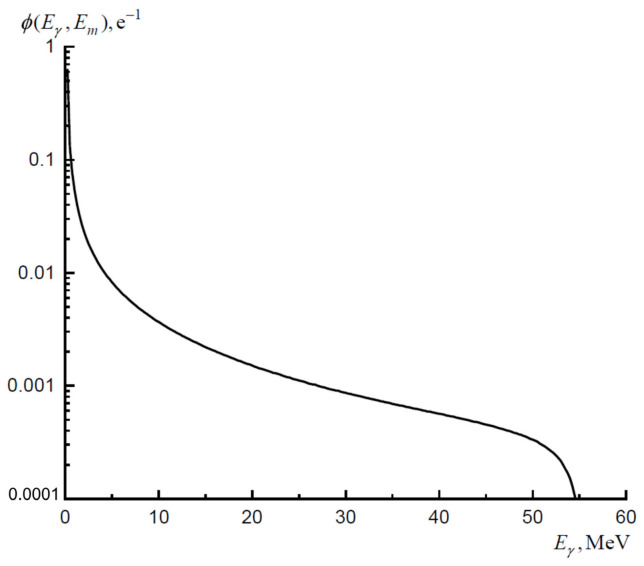
Bremsstrahlung spectrum per one beam electron for used convertors.

The bremsstrahlung spectrum calculated using a full 3D simulation of the irradiation process using the Geant4 program, taking into account the formation of gamma-quanta both in the converter and in the target, is presented on Figure 5.

### 3.2. Irradiation of Targets and Determination of Yields of Photonuclear Reactions

To study the yields of photonuclear reactions on nickel nuclei, two targets were irradiated in RTM-55 microtron with maximum energy of electron beam being 55 MeV [31]. The first target was a plate made of ^nat^Ni with the size of 1 cm × 1 cm, thickness of 500 µm, and weight of 440 mg. Purity of ^nat^Ni was determined in our work by atomic emission spectroscopy using Thermo Scientific ICAP-6500 Duo (Horsham and Loughborough, England) and was 99.78%. The second target made of ^60^Ni purchased from Federal State Unitary Enterprise Combine “Elektrokhimpribor” (Lesnoy, Russia) was thin trapezoidal foil 67 µm thick and with a weight of 86.5 mg. Isotope composition of ^60^Ni-target is presented in Table 2, purity of ^60^Ni—99.9224% according to manufacturer’s data. Tungsten plates 1 mm thick were used as convertors. Monitor targets were a 0.11 mm thick cobalt plates and were located directly after the targets for irradiation. Bremsstrahlung targets, nickel targets, and monitor targets were fully overlapping the beam. Current fluctuations during the irradiations were measured using Faraday cup. Normalization of current was carried out by the processing of bremsstrahlung spectrum and by comparing experimentally measured yield of ^59^Co(γ,n)^58^Co reaction to the yield calculated using known cross-sections. The duration of irradiation of each target was 1 h, average currents were 73 and 48 nA for ^nat^Ni and ^60^Ni accordingly.

The residual activity of nickel targets after irradiation was registered using gamma-ray spectrometer with high-purity germanium detector GC3019 (Canberra Ind, Meridan, CT, USA). Relative efficiency of detector was 30%, energy resolution of detector was 0.9 keV for 122 keV and 1.9 keV for 1.33 MeV. Efficiency calibration of spectrometer was conducted using measurements of activity of certified point sources (^152^Eu, ^137^Cs, ^60^Co, ^241^Am) in different location geometries of source and detector and was also modeled in GEANT4. The selection of the peak maximum in the spectra was carried out using an automatic system for registration and analysis of spectra specially designed for this purpose. Spectra with the duration of 3.5 s each were saved into the database, and the analysis system allowed us to summarize them and display the total spectrum with assigned duration [32]. Activity and yields of the produced isotopes were determined using areas of the most intensive peaks corresponding to the decay of the resulting isotope in spectra of residual activity, taking into account duration of irradiation, duration of transportation and registration of spectrum, and also efficiency of gamma-quanta registration and quantum yield of gamma-transition. Gamma-spectra of each irradiated target were registered three times for 2 days during the 2 months to exactly determine both short-lived and long-lived isotopes.

**Figure 6:**
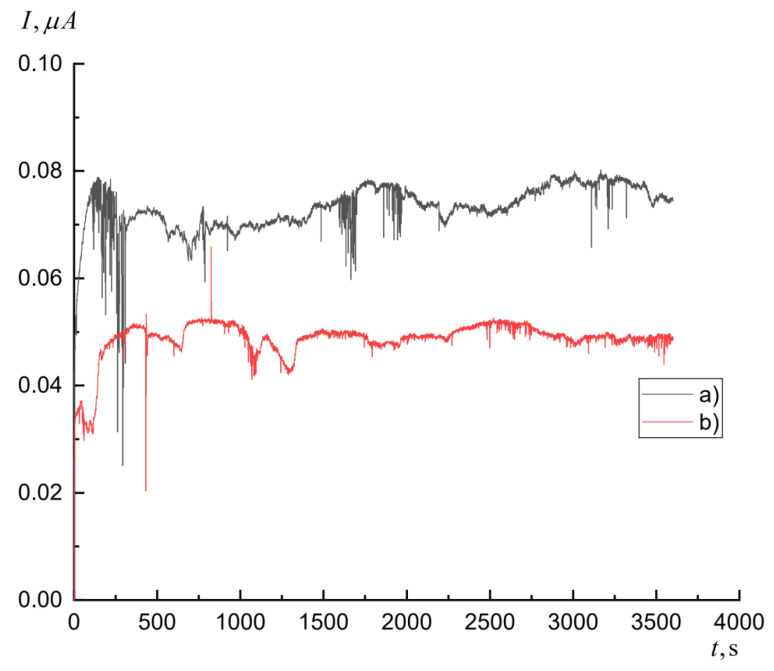
Accelerator current during irradiation of ^nat^Ni (**a**) and ^60^Ni (**b**).

Yields of photonuclear reactions on ^nat^Ni and ^60^Ni in kBq/(µA·h·g/cm^2^), normalized by electron beam charge and surface density of the target, were calculated using Equation (2):

(2) $$Y=\frac{\lambda S}{Ck\rho \left(e^{-\lambda \left(t_{3}-t_{1}\right)}-e^{-\lambda \left(t_{2}-t_{1}\right)}\right)}$$

where *S* is the area of photopeak in spectra of residual activity, corresponding to the gamma-transition during the decay of the resulting nucleus, occurring during the registration, *t*_1_ is the irradiation time, *t*_2_ is the starting time of the registration, *t*_3_ is the ending time of the registration, *λ* is the exponential decay constant, *k* is the coefficient equal to the multiplication of detector efficiency, coefficient of cascade summation, and quantum yield of gamma-quant during gamma-transition, *ρ* is the target surface area, and C is the coefficient taking into account the change in accelerator current during the irradiation (Equation (3), Figure 6).

(3) $$C=\int_{0}^{t_{1}}I\left(t\right)e^{-\lambda t}dt\;$$

Yields of reactions on ^58^Ni were calculated as a difference between the yields of isotope production on natural mix and on ^60^Ni, taking into account the percentage of ^58^Ni and ^60^Ni in natural mix. The accuracy of the selected calculation method was confirmed by the ratio of formation yields after the irradiation of ^60^Ni and ^nat^Ni (3.85 ± 0.05) coincided within the margin of error with the ratio of ^60^Ni content in targets—3.81.

### 3.3. Separation of Co(II) Isotopes from Irradiated Nickel Target

DGA resin Normal (base–N,N,N′,N′-tetraoctyl-1,5-diglycolamide, particle size 100–150 µm, TrisKem Int., Bruz, France) was used for separation. This resin sorbs Co(II) in solutions of HCl with concentration more than 6 M [33]. In these solutions, the distribution coefficient (K_d_) for Co(II) reaches 20, while K_d_(Ni) does not exceed 1 in solutions of HCl with concentration less than 10 M, which allows one to separate Co(II) and Ni(II) using this sorbent. K_d_(Co) decreases with quantity of HCl decreasing: in a more diluted solution of HCl, i.e., less than 4 M, Co(II) is not retained on the column.

To optimize the separation method of carrier-free Co(II) from irradiated nickel, experiments with the plate made of ^nat^Ni (1 cm × 1 cm), similar to the plate from Section 3.2 were carried out. The irradiated plate was dissolved in 12 M HCl by prolonged heating, then the solution was evaporated to dryness in 9 M HCl. The sorbent was preliminarily held in 0.001 M HCl for 1 h, then the column (height 4 cm, diameter 0.6 cm, volume 2 mL) was filled with it. After the dissolution of the irradiated target, 0.5–1 mL of obtained solution was placed in the column with DGA resin; Co(II), unlike Ni(II), was sorbed onto the column. The remaining Ni(II) was eluted with 5 mL of 9 M HCl, then Co(II) was eluted with 5 mL of HCl solution with the concentration varying from 0.01 M to 3 M. Fractions of 1 mL were collected during the separation, the content of Co(II) and Ni(II) was determined using gamma-spectroscopy with high-purity germanium detector GC1020 (Canberra Ind.). Co(II) was identified by peaks of ^57^Co (122 keV, 85.6%), and Ni(II) was identified by peaks of ^57^Ni (127 keV, 16.7%) and ^56^Ni (158 keV, 98.8%).

## 4. Conclusions

Targets made of ^nat^Ni and ^60^Ni were irradiated by bremsstrahlung photons with energy up to 55 MeV; the radionuclide composition and yields of nuclear reactions on ^nat^Ni, ^60^Ni, and ^58^Ni were determined. It was established that in every case, the activities of produced ^56,57,58^Co were higher than the activity of ^55^Co, and therefore enough for preclinical research of radiopharmaceuticals based on cobalt. It was also demonstrated that the radionuclide purity of ^55^Co produced by the photonuclear method at 55 MeV is not sufficient for PET. A fast, simple, and effective method of cobalt isotopes separation without a carrier from irradiated targets by extraction chromatography was developed; it was demonstrated that the separation of Co(II) is possible in wide range of HCl concentrations (from 0.01 to 3 M). The separation factor of Ni/Co was 2.8·10^5^, the yield of Co(II) was close to quantitative, and separation lasted for no longer than 0.5 h.

## Figures and Tables

**Table 1:** Yields of photonuclear reactions on ^nat^Ni, ^60^Ni (obtained experimentally), and ^58^Ni (calculated using yields on ^nat^Ni, ^60^Ni nuclei) with maximum energy of bremsstrahlung photons being 55 MeV. Values obtained by TALYS [29] are presented in brackets.

Isotope	T_1/2_	Y_EOB_, kBq/(µA·h·g/cm^2^)		
		^nat^Ni	^60^Ni	^58^Ni
^56^Ni	6.08 d	20.7 ± 0.3 (32.5)	0.16 ± 0.01 (0.06)	29.6 ± 0.3 (47.7)
^57^Ni	35.6 h	3883 ± 76 (4404)	48.3 ± 1.0 (21)	5440 ± 93 (6460)
^55^Co	17.53 h	60.2 ± 4.2 (72.9)	1.1 ± 0.1 (0.02)	82.4 ± 5.1 (107)
^56^Co	77.27 d	16.5 ± 0.1 (17.4)	0.48 ± 0.03 (0.16)	21.6 ± 0.2 (25.4)
^57^Co	271.8 d	66.1 ± 0.4 (46.5)	2.75 ± 0.04 (1.35)	81.8 ± 0.6 (67.8)
^58^Co	70.86 d	10.3 ± 0.1 (3.6)	39.6 ± 0.4 (13.6)	0

**Table 2:** Isotope composition of ^60^Ni foil according to manufacturer’s data.

Isotope	Content, %
^58^Ni	0.31
^60^Ni	99.6 ± 0.1
^61^Ni	0.05
^62^Ni	0.04
^64^Ni	<0.05

## Data Availability

Not applicable.
